# Supplementary material for: Sparse estimation of mutual information landscapes quantifies information transmission through cellular biochemical reaction networks
Source: Commun Biol. 2020 Apr 30;3:203. doi: 10.1038/s42003-020-0901-9 (PMC7192899; doi:10.1038/s42003-020-0901-9)
Supplement: Supplementary file 1 — Supplementary Information [file 42003_2020_901_MOESM1_ESM.pdf]

Supplementary Information:

Supplementary Figures

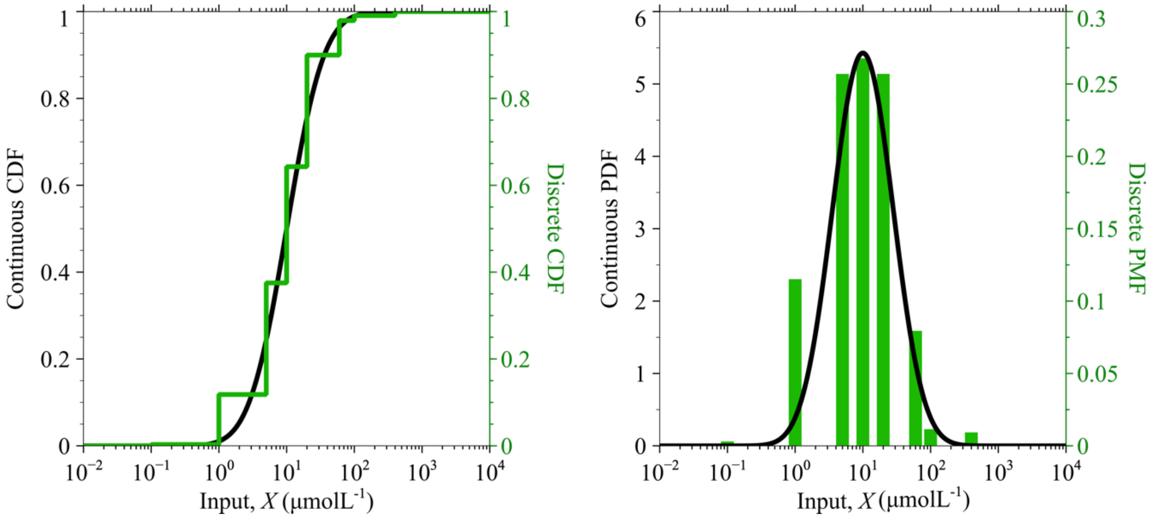

Supplementary Figure 1: Example of a stochastic reduced-order model. (a) The black line is the continuous CDF of a beta distribution *p(X)*. The green stepped line is the discrete CDF of the stochastic-reduced order approximation obtained by solving Eq. (7). (b) The black line is the continuous PDF of the same beta distribution shown in (a), and the PMF, $P(X = x_k)$, of the best discrete approximation is shown in green.

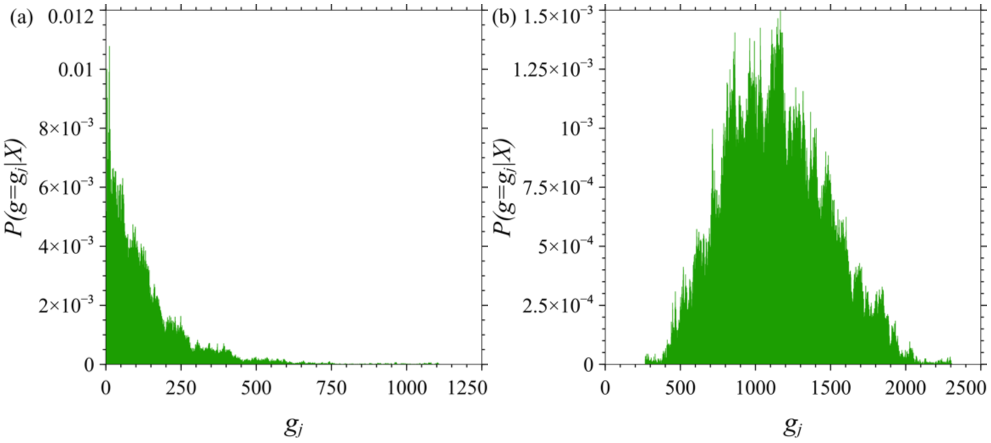

Supplementary Figure 2: YFP distribution from the simulated BRN for two different input values or IPTG concentrations: (a) output for X = 10 μmolL^{-1}, and (b) output for X = 100 μmolL^{-1}.

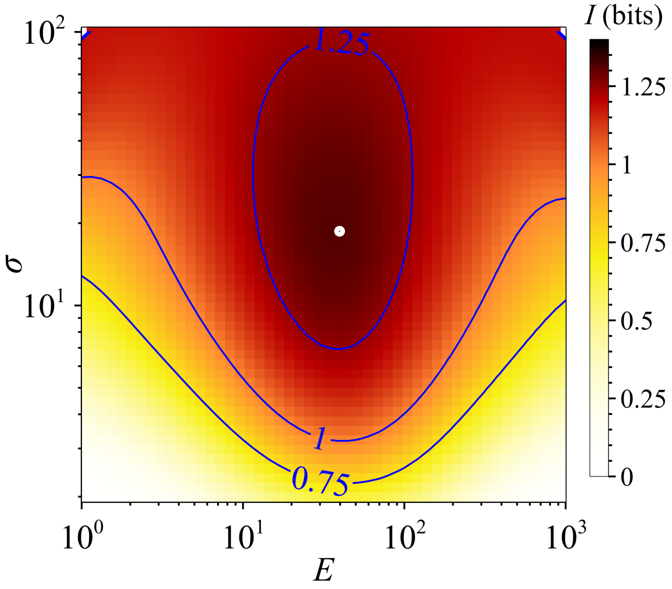

Supplementary Figure 3: Correct mutual information landscape (I_{correct} for Fig. 2 in main text) for simulated BRN computed by numerical integration of Eq. (12).

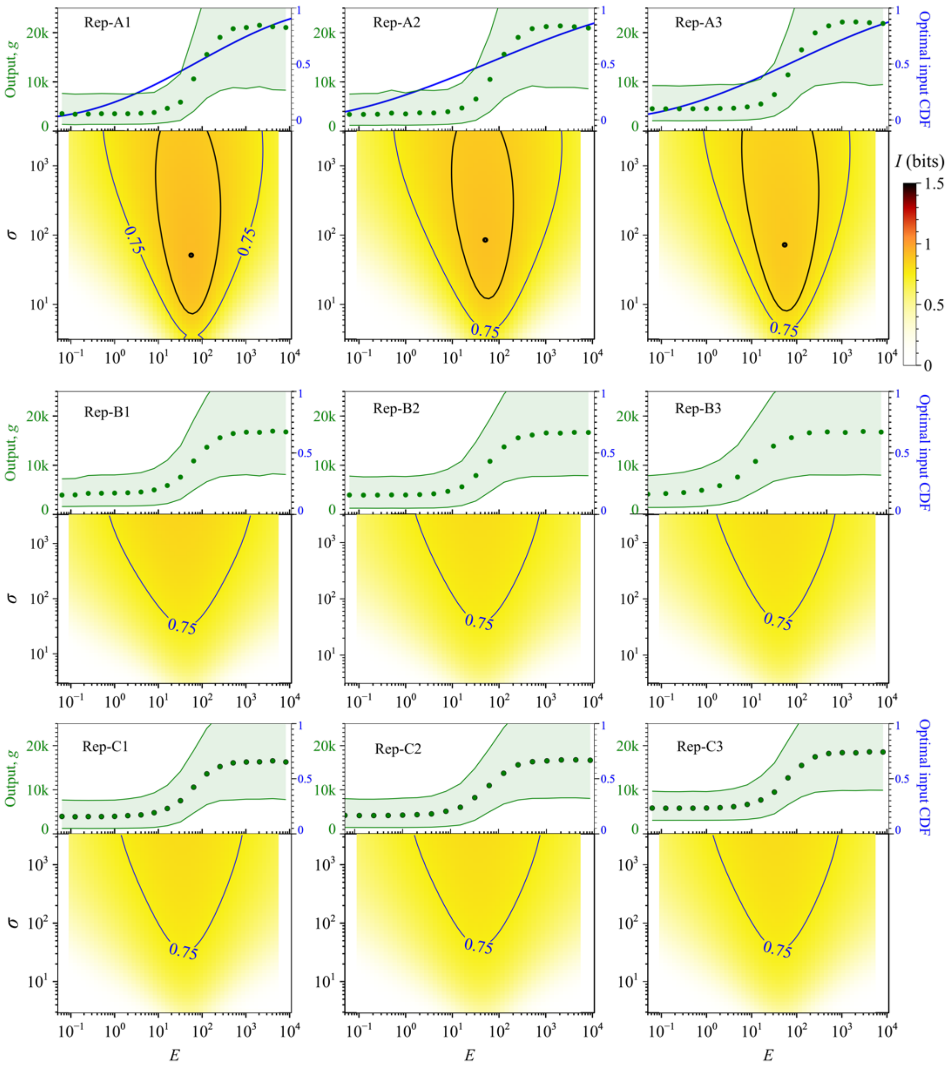

Supplementary Figure 4: Response functions, mutual information landscapes, and optimal input distributions for biological and technical replicates of BRNs with deactivated *lacY* and relative *lacI* translation rate of 0.008 (Fig. 4b in main text).

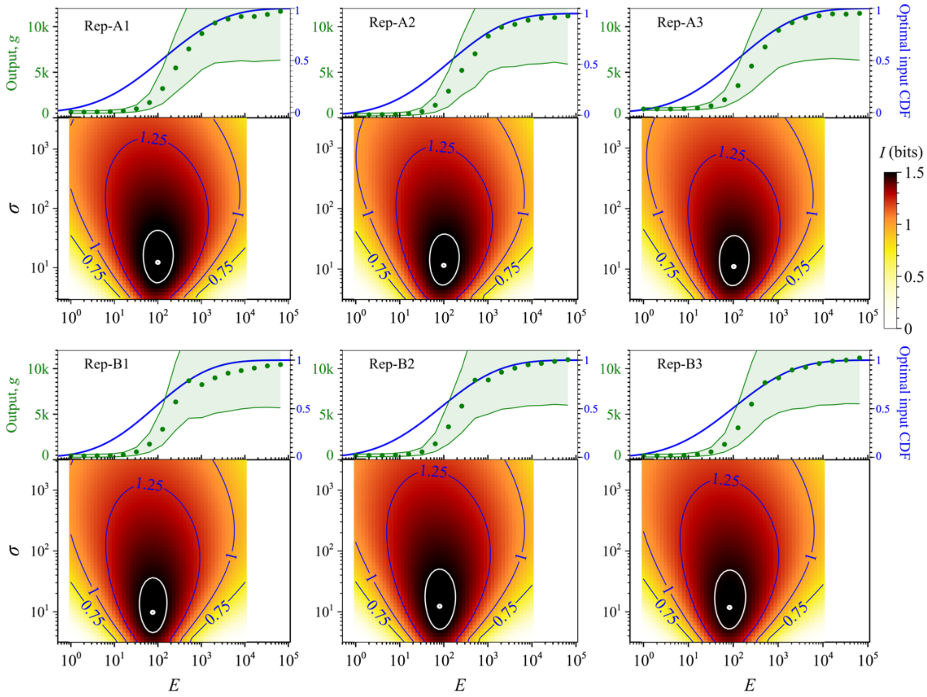

Supplementary Figure 5: Response functions, mutual information landscapes, and optimal input distributions for biological and technical replicates of BRNs with deactivated *lacY* and relative *lacI* translation rate of 1 (Fig. 4c in main text).

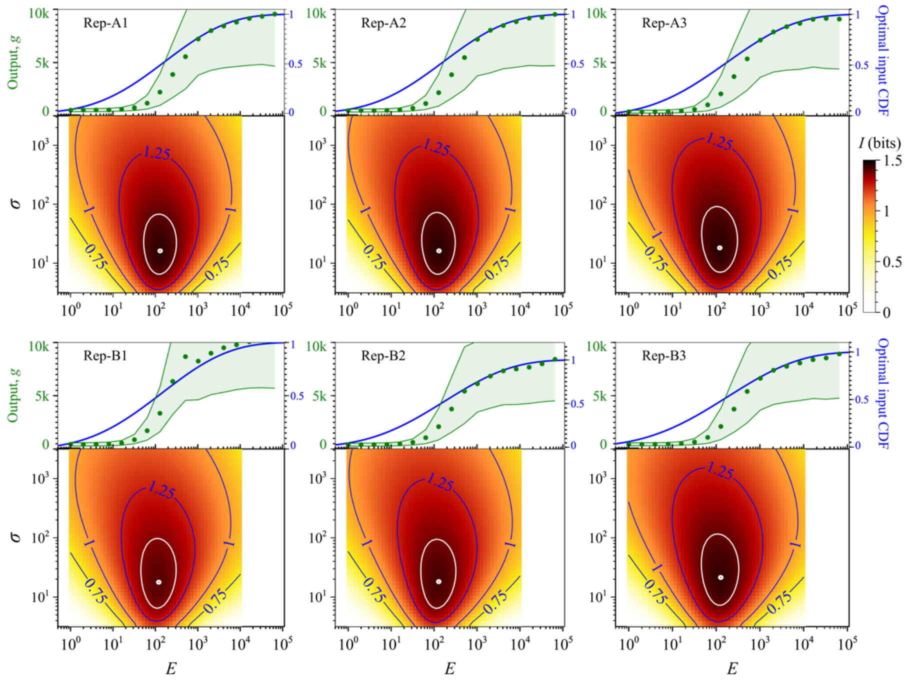

Supplementary Figure 6: Response functions, mutual information landscapes, and optimal input distributions for biological and technical replicates of BRNs with deactivated *lacY* and relative *lacI* translation rate of 1 (Fig. 4c in main text).

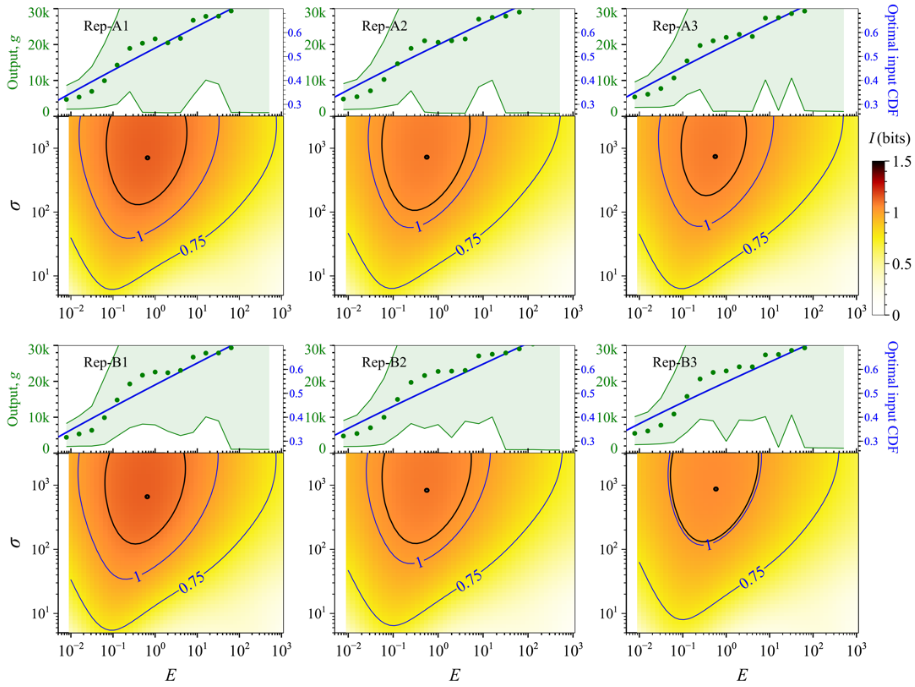

Supplementary Figure 7: Response functions, mutual information landscapes, and optimal input distributions for biological and technical replicates of BRNs with activated *lacY* and relative *lacI* translation rate of 0.008 (Fig. 4e in main text).

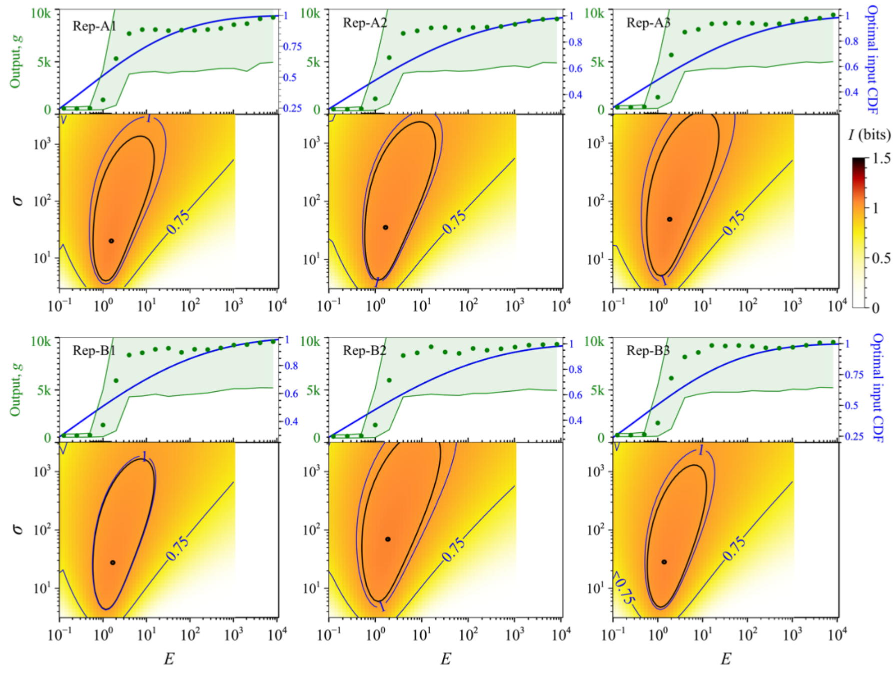

Supplementary Figure 8: Response functions, mutual information landscapes, and optimal input distributions for biological and technical replicates of BRNs with activated *lacY* and relative *lacI* translation rate of 1 (Fig. 4f in main text).

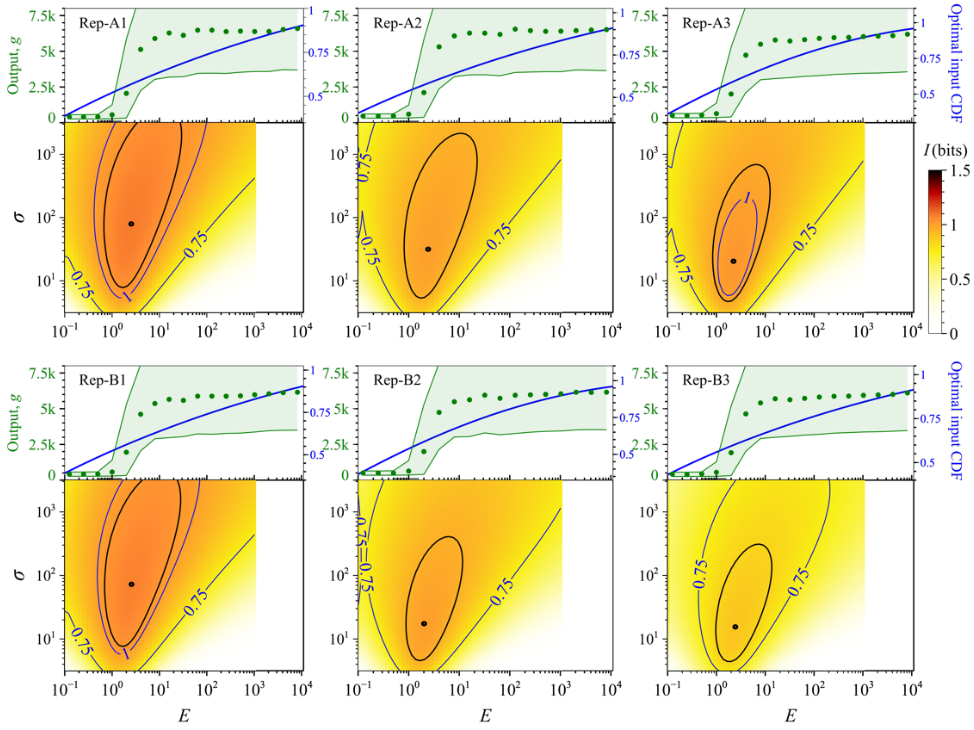

Supplementary Figure 9: Response functions, mutual information landscapes, and optimal input distributions for biological and technical replicates of BRNs with activated *lacY* and relative *lacI* translation rate of 10 (Fig. 4g in main text).

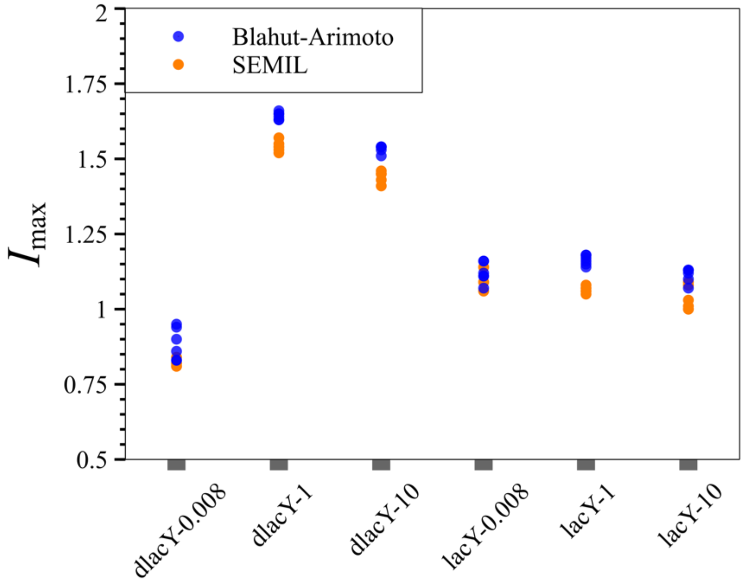

Supplementary Figure 10: Comparison of maximum mutual information from Blahut-Arimoto algorithm and SEMIL for each of the six experimentally-measured BRNs (Supplementary Figure 5-10).

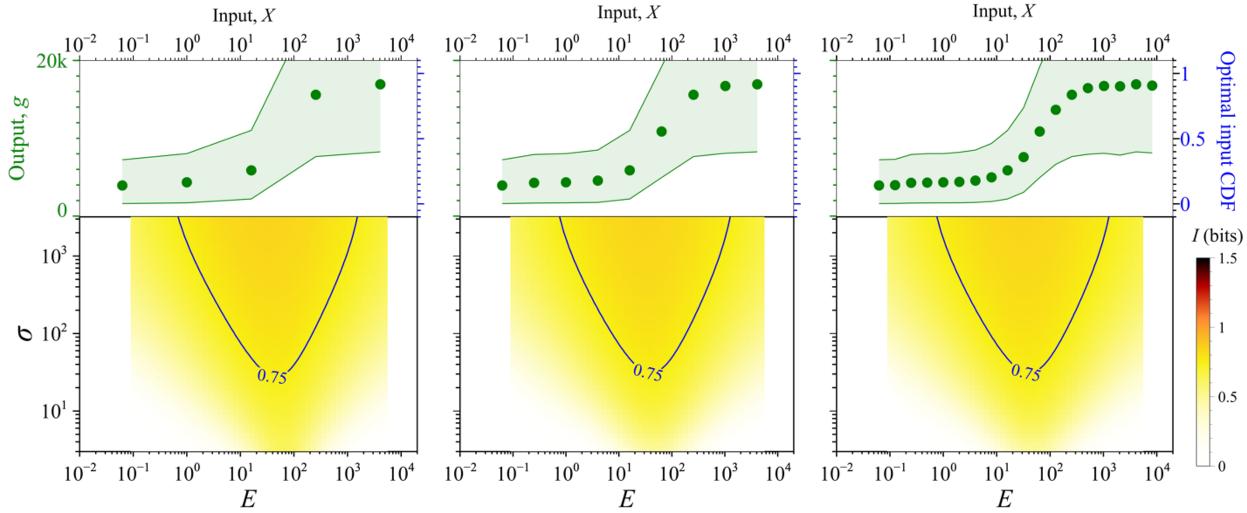

Supplementary Figure 11: Response functions and mutual information landscapes for replicate B1 of BRNs with deactivated *lacY* and relative *lacI* translation rate of 0.008 (Supplementary Figure 5). The leftmost panel uses one-fourth of the output data, the middle panel uses half of the input data, and the rightmost panel uses the total dataset.

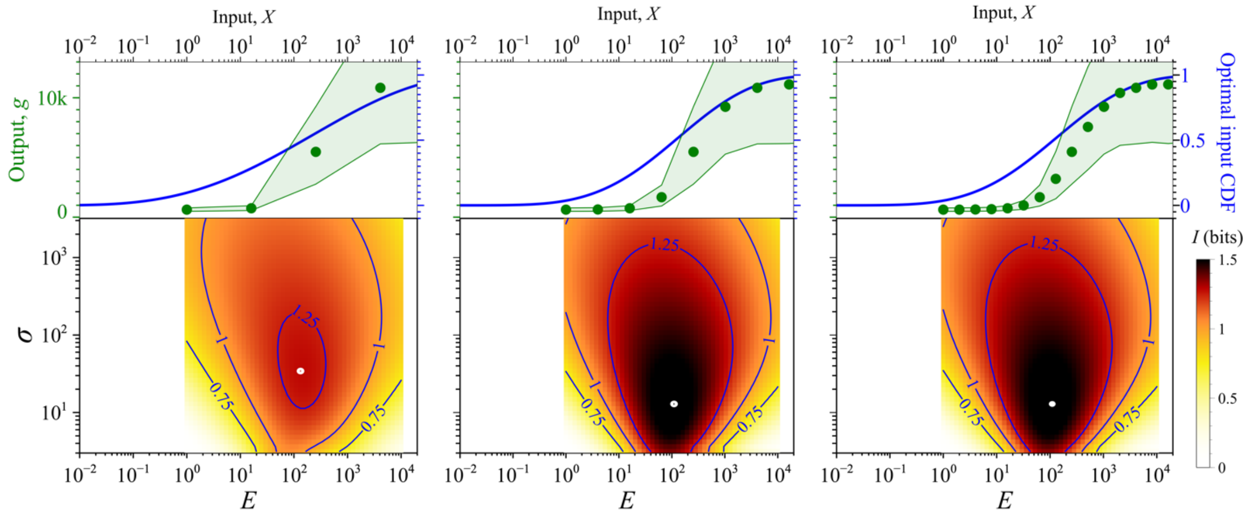

Supplementary Figure 12: Response functions, mutual information landscapes, and optimal input distribution for replicate A1 of BRNs with deactivated *lacY* and relative *lacI* translation rate of 1 (Supplementary Figure 6). The leftmost panel uses one-fourth of the output data, the middle panel uses half of the input data, and the rightmost panel uses the total dataset.

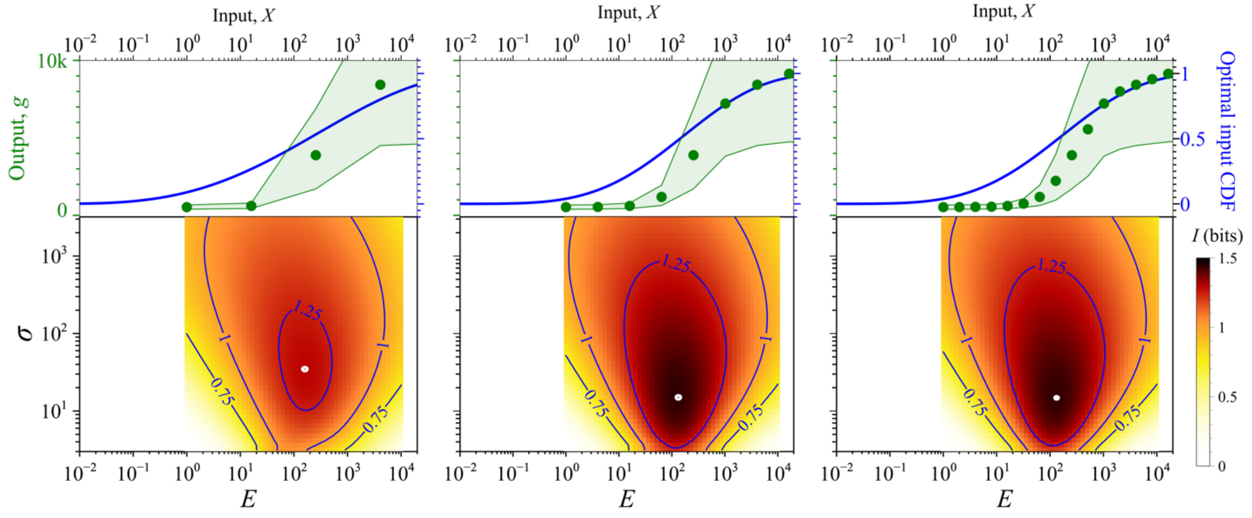

Supplementary Figure 13: Response functions, mutual information landscapes, and optimal input distribution for replicate A1 of BRNs with deactivated *lacY* and relative *lacI* translation rate of 10 (Supplementary Figure 7). The leftmost panel uses one-fourth of the output data, the middle panel uses half of the input data, and the rightmost panel uses the total dataset.

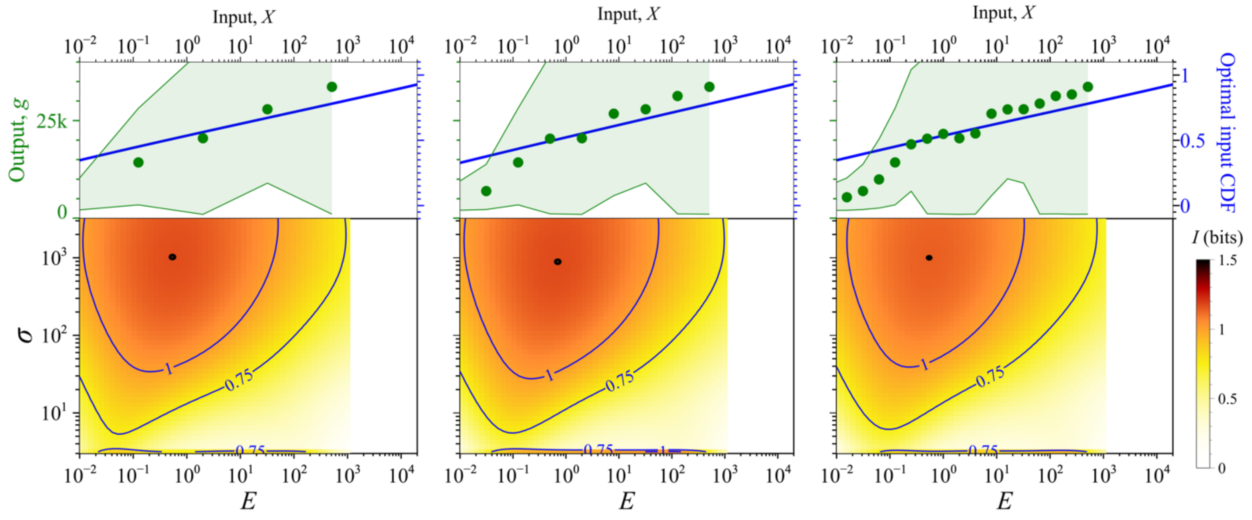

Supplementary Figure 14: Response functions, mutual information landscapes, and optimal input distribution for replicate A1 of BRNs with activated *lacY* and relative *lacI* translation rate of 0.008 (Supplementary Figure 8). The leftmost panel uses one-fourth of the output data, the middle panel uses half of the input data, and the rightmost panel uses the total dataset.

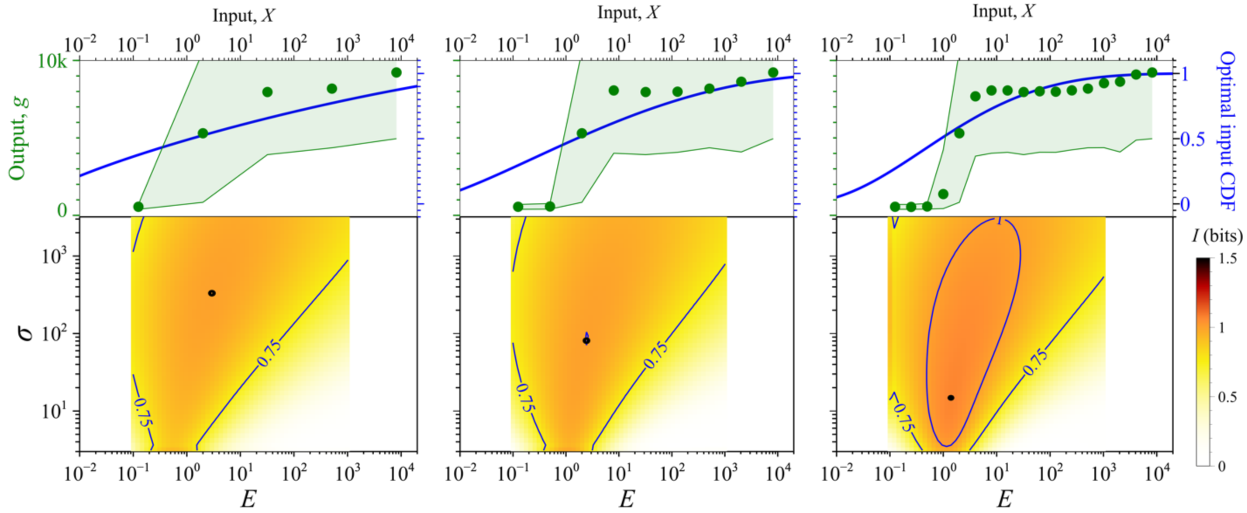

Supplementary Figure 15: Response functions, mutual information landscapes, and optimal input distribution for replicate A1 of BRNs with activated *lacY* and relative *lacI* translation rate of 1 (Supplementary Figure 9). The leftmost panel uses one-fourth of the output data, the middle panel uses half of the input data, and the rightmost panel uses the total dataset.

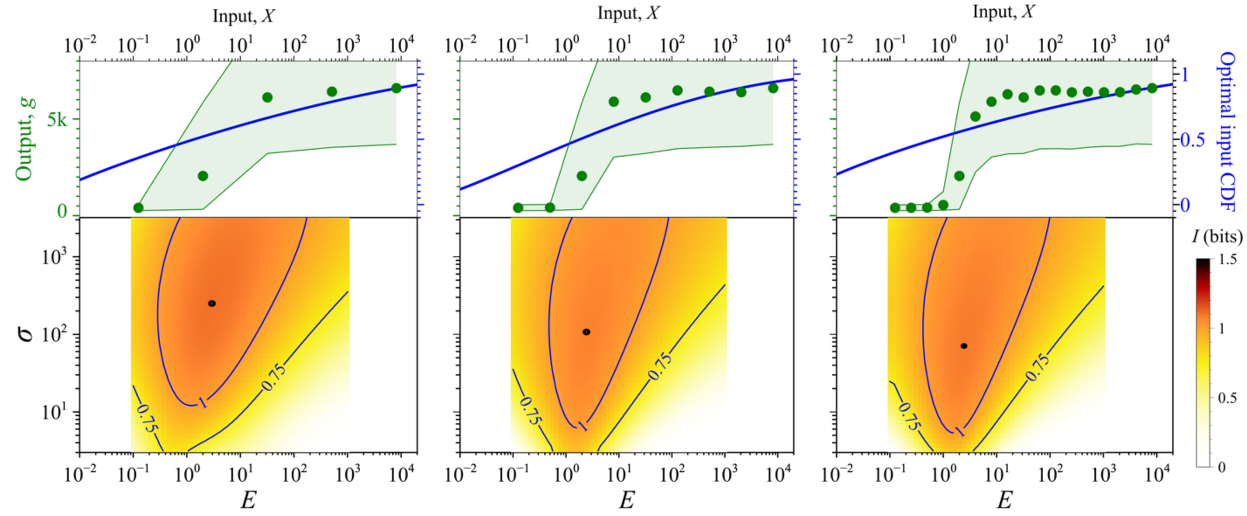

Supplementary Figure 16: Response functions, mutual information landscapes, and optimal input distribution for replicate A1 of BRNs with activated *lacY* and relative *lacI* translation rate of 10 (Supplementary Figure 10). The leftmost panel uses one-fourth of the output data, the middle panel uses half of the input data, and the rightmost panel uses the total dataset.

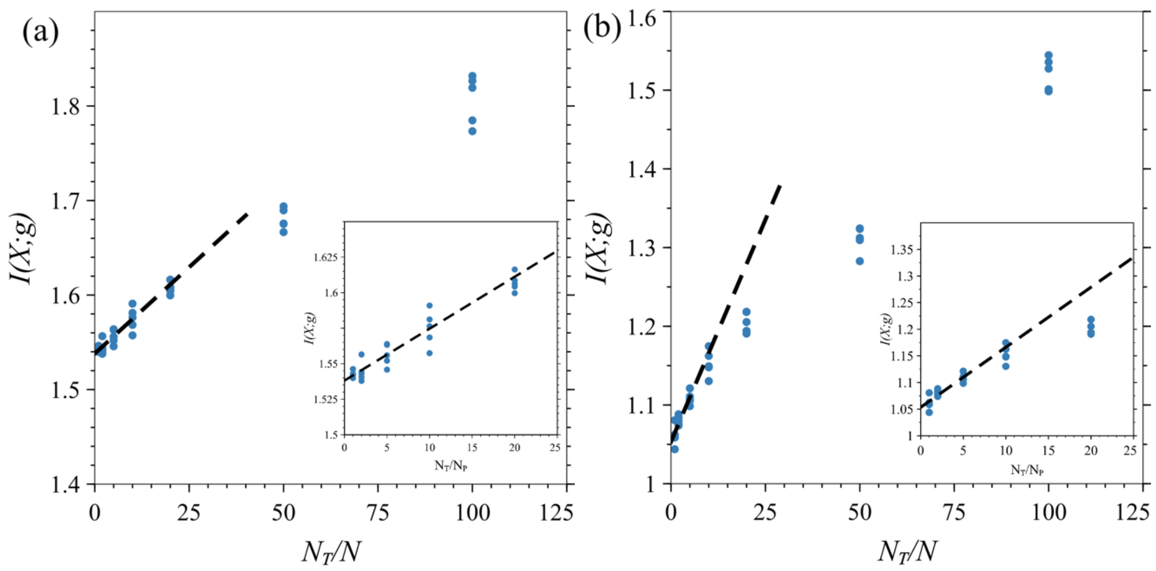

Supplementary Figure 17: Examples for correction of finite-sampling bias. With a smaller sample size N, the mutual information, $I(X;g)$, is overestimated by a higher amount. The Y-intercept of the linear fit for multiple subsample sizes was used to estimate the unbiased mutual information from finite data. We used this method to compute the limiting mutual information for the entire design space for all the engineered BRNs. (a) for BRN with deactivated *lacY* and relative *lacI* translation rate constant 1, replicate B1, $I = 1.54 + 0.00174(N_T/N)$, and (b) for BRN with *lacY* and relative *lacI* translation rate constant 1, replicate A1, $I = 1.053 + 0.011(N_T/N)$. Each of the plots shows the mutual information values at the optimal input distribution in the design space for the respective BRNs.

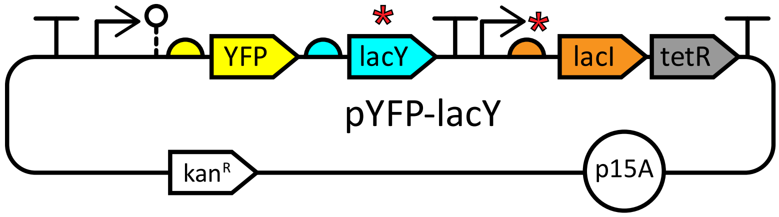

Supplementary Figure 18: Plasmid map of construct used for flow cytometry measurements. For data without feedback (main text Fig. 4 panels b, c, d), three sequential codons in the *lacY* CDS were mutated to stop codons (positions D37, I38, and N39, indicated with red asterisk above cyan *lacY* symbol). Additionally, to modify the *lacI* protein concentration in the cell, the RBS of *lacI* was changed to modify the translation rate of *lacI*, as indicated with red asterisk above *lacI* RBS. Sequences described below.

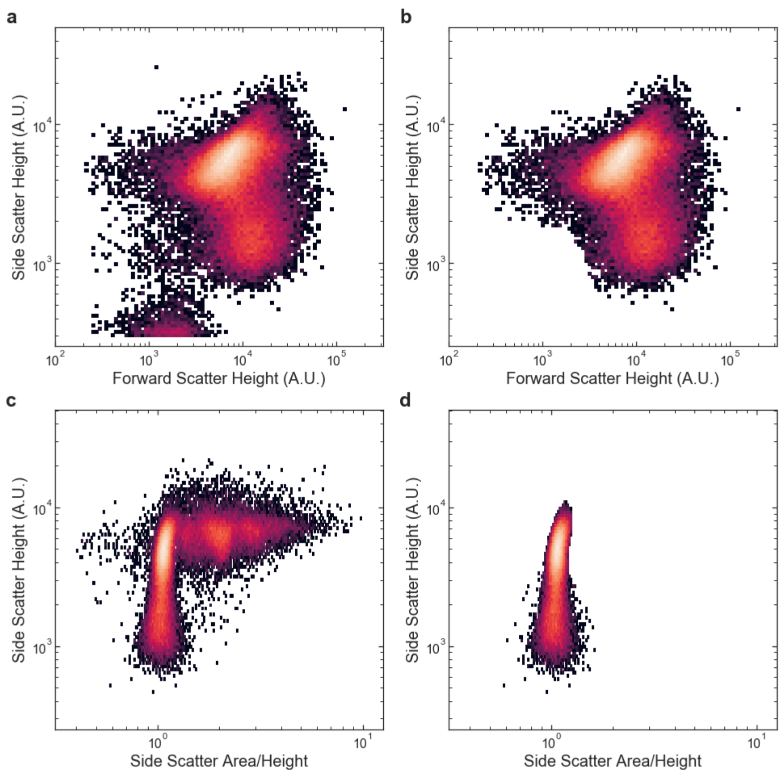

Supplementary Figure 19: Example of automated gating used for cytometry analysis. **a**, Side scatter signal vs. forward scatter signal before automated cell gating. **b**, Side scatter signal vs. forward scatter signal after automated cell gating. **c**, Side scatter signal vs. side scatter area/height ratio before automated singlet gating. **d**, Side scatter signal vs. side scatter area/height ratio after automated singlet gating. A blank (buffer only) sample was measured before the *E. coli* samples, and was used to automatically determine the gate to apply to the side scatter vs. forward scatter data to discriminate cell events from non-cell events (**a, b**). The Attune cytometer is calibrated so that the scattering area and height parameters are equal for singlet events. A second automated gating algorithm took advantage of this to select singlet cells based on the identification of detection events with side scatter area/height ≅ 1.

Supplementary Tables

Symbol	Chemical species
M_R	*lacI* repressor mRNA
R	*lacI* repressor monomer
R_2	*lacI* repressor dimer
O	*lacO* operator
R_2O	Repressor-operator complex
I	Intracellular IPTG
IPTG	Extracellular IPTG
I_2R_2	Repressor-inducer complex
Φ	Generic source or sink
MYFP	Output protein mRNA

YFP	Output protein

Supplementary Table 1: Components for the simple BRN model used for Gillespie simulation-based validation of SEMIL.

Reaction	
1	$\Phi \xrightarrow{k_{sMR}} M_R$
2	$M_R \xrightarrow{k_{sR}} M_R + R$
3	$2R \underset{k_{-2R}}{\overset{k_{2R}}{\rightleftharpoons}} R_2$
4	$R_2 + O \underset{k_{-r}}{\overset{k_r}{\rightleftharpoons}} R_2O$
5	$2I + R_2 \underset{k_{-dr1}}{\overset{k_{dr1}}{\rightleftharpoons}} I_2R_2$
6	$2I + R_2O \underset{k_{-dr2}}{\overset{k_{dr2}}{\rightleftharpoons}} I_2R_2 + O$
7	$IPTG \underset{k_{-t}}{\overset{k_t}{\rightleftharpoons}} I$
8	$O \xrightarrow{k_{sM}} O + MYFP$
9	$R_2O \xrightarrow{k_{s0M}} R_2O + MYFP$
10	$MYFP \xrightarrow{k_{sYFP}} MYFP + YFP$
Degradation reactions:	
11	$M_{(i)} \xrightarrow{k_{d(i)}} \Phi \quad i \in \{\text{all proteins and mRNAs}\}$

Supplementary Table 2: Reactions of the simple BRN model used for Gillespie simulation-based validation of SEMIL.

Reaction	Forward	Reverse
1	$V_{E.coli} \cdot N_A \cdot k_{sMR}$	
2	$k_{sR} \cdot \mathrm{M_R}$	
3	$\frac{k_{2R}}{2V_{E.coli} \cdot N_A} \cdot \mathrm{R} \cdot (\mathrm{R} - 1)$	$k_{-2R} \cdot \mathrm{R_2}$
4	$\frac{k_r}{V_{E.coli} \cdot N_A} \cdot \mathrm{R_2} \cdot \mathrm{O}$	$k_{-r} \cdot \mathrm{R_2O}$
5	$\frac{k_{dr1}}{2(V_{E.coli} \cdot N_A)^2} \cdot \mathrm{I} \cdot (\mathrm{I} - 1) \cdot \mathrm{R_2}$	$k_{-dr1} \cdot \mathrm{I_2R_2}$
6	$\frac{k_{dr2}}{2(V_{E.coli} \cdot N_A)^2} \cdot \mathrm{I} \cdot (\mathrm{I} - 1) \cdot \mathrm{R_2O}$	$\frac{k_{-dr2}}{V_{E.coli} \cdot N_A} \cdot \mathrm{I_2R_2} \cdot \mathrm{O}$
7	$k_t \cdot \mathrm{IPTG}$	$k_{-t} \cdot \mathrm{I}$
8	$k_{sM} \cdot \mathrm{O}$	
9	$k_{s0M} \cdot \mathrm{R_2O}$	
10	$k_{\mathrm{sYFP}} \cdot \mathrm{MYFP}$	
11	$k_{d(i)} \cdot \mathrm{M_{(i)}}$	

Supplementary Table 3: Propensity function of the reactions in the simple BRN model used for Gillespie
simulation-based validation of SEMIL.

Reaction	Rate parameter	Value
1	k_{sMR}	$0.23\,\mathrm{nmolL^{-1}min^{-1}}$
2	k_{sR}	$15\,\mathrm{min^{-1}}$
3	k_{2R}	$50\,\mathrm{nmol^{-1}Lmin^{-1}}$
	k_{-2R}	$10^{-3}\,\mathrm{min^{-1}}$
4	k_r	$9.6\,\mathrm{nmol^{-1}Lmin^{-1}}$
	k_{-r}	$2.4\,\mathrm{min^{-1}}$
5	k_{dr1}	$3 \times 10^{-7}\,\mathrm{nmol^{-2}L^2min^{-1}}$
	k_{-dr1}	$12\,\mathrm{min^{-1}}$
6	k_{dr2}	$3 \times 10^{-7}\,\mathrm{nmol^{-2}L^2min^{-1}}$
	k_{-dr2}	$4.8 \times 10^3\,\mathrm{nmol^{-1}Lmin^{-1}}$
7	k_t	$0.92\,\mathrm{min^{-1}}$
	k_{-t}	$0.92\,\mathrm{min^{-1}}$
8	k_{sM}	$0.5\,\mathrm{min^{-1}}$
9	k_{s0M}	$0.01\,\mathrm{min^{-1}}$
12	k_{sYFP}	$30\,\mathrm{min^{-1}}$
13	$k_{d(i)}$	$0.462\,\mathrm{min^{-1}}$ for mRNAs
	$k_{d(i)}$	$0.2\,\mathrm{min^{-1}}$ for proteins

Supplementary Table 4: Rate constants of the reactions in the simple BRN model used for Gillespie simulation-based validation of SEMIL.

Relative lacI Translation Rate	Sequence
1	GGAAGAGAGTCAATTCAGGGTGGTGAAT
10	GGAAGAGAGTCAATAGGAGGTGGTGAAT
0.008	GCTAACTGTCTCATTCTCCCTCCTGAAT

Supplementary Table 5: RBS sequence modifications for changing the *lacI* translation rate.

Category	Factor	Level
Media components	KH_2PO_4	3 gL^{-1}
	Na_2HPO_4	6.78 gL^{-1}
	NaCl	0.5 gL^{-1}
	NH_4Cl	1.0 gL^{-1}
	D – glucose	4.0 gL^{-1}
	Casamino acids	2.0 gL^{-1}
	Casamino acids source	Fisher Scientific, catalog number BP1424-500
	$CaCl_2$	0.1 gL^{-1}
	Mg_2SO_4	0.493 gL^{-1}
	Vitamin B_1	0.34 gL^{-1}
	Water	DI water (18 MΩ-cm)

Supplementary Table 6: Details of *E.coli* Culture Conditions.

Category(contd.)	Factor	Level (Overnight growth)	Level (outgrowth)
Container geometry	Type	culture tube	96-well plate
	Well shape	Round	Square
	Well bottom	Round	Pyramid-flat
	Well volume	14 mL	1.1 mL
	Fill volume	14 % (2 mL)	45 % (0.5 mL)
	Cover	Snap cap	Gas permeable clear heat seal with slits[a]
Container shaking	Shaking speed	460 rpm	269 rpm
	Shaking speed	12.5 mm	6 mm
	Shaking model	Orbital	Double orbital
Time	Growth Time	48 h	3h:35m
Environment	Temperature	37 °C	37 °C
	Relative humidity	not controlled	not controlled
Selective agents	Antibiotic type	Kanamycin	Kanamycin
	Antibiotic concentration	50 $\mu g\ ml^{-1}$	50 $\mu g\ ml^{-1}$
Inoculum	Type	Single colony	Overnight culture
	Concentration at inoculation	N/A	1000-fold diluted from overnight culture[b]
	Age of inoculum at inoculation	16 h	16 h
Inducers	IPTG	0	0 to 2.048 $mmolL^{-1}$

[a] Brooks Life Sciences part number 4ti-0541/SLIT

[b] Overnight culture was diluted into the 96-well plate in three steps, with 10-fold dilution at each step

Supplementary Table 7: Details of *E.coli* Culture Conditions.

Supplementary Methods

1. **STOCHASTIC REDUCED ORDER MODEL OF INPUT DISTRIBUTION**

SEMIL is used to estimate the mutual information landscape of a BRN across a design space of possible input probability distributions. This is accomplished by first computing a discrete approximation of the continuous input distribution, $p(X)$, for every point in the design space. This discrete approximation is obtained using stochastic reduced-order modeling as described below.

Consider a stochastic input variable X with probability distribution $p(X)$ over a support $[X_{min}, X_{max}]$. Let a trial discrete approximation be a set of input values $\{x_k\}$ and associated probability masses $P^*(X = x_k)$, such that $\sum_k P^*(X = x_k) = 1$. The cumulative distribution function (CDF) for the continuous input variable is

$$F(X) = \int_{X_{min}}^{X} p(x)\,dx \tag{1}$$

and the CDF for the discrete approximation is

$$F^*(X) = \sum_k P^*(X = x_k)\Theta(X - x_k) \tag{2}$$

where $\Theta(X - X_k)$ is the Heaviside step function, i.e. $\Theta(a) = 1$ when $a \geq 0$ and $\Theta(a) = 0$ when $a < 0$. The nth moment of the continuous input variable is

$$\mu(n) = \int_{X_{min}}^{X_{max}} X^n p(X)\,dX \tag{3}$$

and the same moment for the discrete approximation is

$$\mu^*(n) = \sum_k x_k^n P^*(X = x_k) \tag{4}$$

We define two error terms due to the differences between the continuous CDFs and moments and their respective discrete approximations:

$$\epsilon_F(p, P^*) \equiv \frac{1}{2}\int_{X_{min}}^{X_{max}} (F(X) - F^*(X))^2\,dX \tag{5}$$

and

$$\epsilon_\mu(p, P^*) \equiv \frac{1}{2}\sum_{i=1}^{m}(\mu(i) - \mu^*(i))^2 \quad (6)$$

where *m* is the total number of moments over which we are quantifying the error ϵ_μ. For all the SEMIL calculations presented in this work we used $m = 2$. Using the two error functions (5) and (6), we can define the optimal probability masses, $\{P(X = x_k)\}$ as the solution to the minimization problem:

$$\{P(X = x_k)\} \equiv \arg\min_{P^*}(\alpha_F \epsilon_F(p, P^*) + \alpha_\mu \epsilon_\mu(p, P^*))$$
$$\text{such that} \sum_k P^*(X = x_k) = 1$$
$$\text{and } P^*(X = x_k) \geq 0 \text{ for all } k \quad (7)$$

where α_F and α_μ are factors to control the relative importance of the CDF error and moment error in the total optimization error functional. Supplementary Figure 1 shows an example of an optimal discrete approximation of a continuous distribution of the input variable. The set of values for (α_F , α_μ) were chosen by trying out various combinations. Increasing the ratio of α_F/α_μ improved the accuracy of the stochastic reduced-order model for input distributions with high geometric standard deviation but decreased it for low geometric standard deviation. For all the SEMIL calculations presented in this work we used $\alpha_F = 100$ and $\alpha_\mu = 1$ which resulted in a similar magnitude of error for both high and low geometric standard deviation input distributions.

In previous work with stochastic reduced-order models [1], the values of the inputs used in the discrete input set, x_k, were allowed to vary during the process of minimizing $\epsilon_F(p,P^*)$ and $\epsilon_\mu(p,P^*)$. Under this condition the solution to Eq. (7) not only yields the best probability masses $P(X = x_k)$, but also the best input values $\{x\}$ for those masses, and the error from sparse approximation decreases monotonically as the number of inputs is increased [1]. However, to apply this scheme for experimentally measured BRNs would require measurement of the output response data at a new set of input levels (or inducer concentrations) for every input distribution in the design space. Hence, to enable application with experimental data we keep the input values $\{x_k\}$ fixed.

2. VALIDATION OF SEMIL WITH ANALYTICAL BRN MODEL

To test the validity and quantitative accuracy of SEMIL, we used simulated data from an analytical model of BRN output. For this, we chose a model BRN response function where each value of the input, *X*, results in a gamma distribution of the output *g*.

$$p(g|X) \equiv f(g; k(X), \theta(X)) = \frac{g^{k-1}e^{-g/\theta}}{\theta^k \Gamma(\theta)} \quad (8)$$

where $\Gamma(\cdot)$ is the Gamma function, k is the shape parameter of the gamma distribution, and θ is the scale parameter of the gamma distribution.

Based on fits to real BRN output data, we used the Hill equation as an analytical model for the input dependence of the shape and scale parameters of the output gamma distribution.

$$k = 9\frac{X^m}{X^m + \mathrm{IC}_{50,1}^m} + 1 \tag{9}$$

and

$$\theta = 9\frac{X^n}{X^n + \mathrm{IC}_{50,2}^n} + 1 \tag{10}$$

For the set of calculations presented in Fig. 2(a)-(c) in the main text, we chose $m = n = 2$ and $\mathrm{IC}_{50,1} = \mathrm{IC}_{50,2} = 50$.

To check the accuracy of SEMIL and to determine how that accuracy depends on the number of discrete input values used, we used SEMIL to estimate mutual information for the model BRN using two sets of input values: {2, 8, 32, 128, 512}, and {2, 4, 8, 16, 32, 64, 128, 256, 512, 1024}. For each of the input values we determined k and θ from Eq. (9)-(10), and generated mock data by drawing 10,000 samples from the resulting gamma distribution, Eq. (8).

We first used SEMIL to determine the input distribution at which the mutual information is maximum, or the optimal input distribution, $p_C(X)$, for this model response function. The geometric mean and the geometric standard deviation of $p_C(X)$, were $E = 50.1$ and $\sigma = 2.51$, respectively. Then we used SEMIL to estimate mutual information, $I(X;g)$, for two sets of input distributions: (1) with fixed $E = 50.1$ and $\sigma \in [1,10^3]$, and (2) input distributions with fixed $\sigma = 2.51$ and $E \in$ 2, 5×10^2].

To obtain the correct mutual information (true solution) for the same sets of input distributions, we numerically integrated the continuous form of the mutual information, using $p(g|X)$ as given in Eq. (11):

$$I(X;g) = \int_X p(X) \int_g p(g|X) \log_2 \frac{p(g|X)}{p(g)} \, dg \, dX \tag{11}$$

3. COMPARISON OF SEMIL WITH RESULTS FOR THE SMALL NOISE LIMIT

Validating SEMIL with a model BRN response function for which the analytical solution to the optimal input distribution is known is ideal. Such analytical results are rare for biological response

functions. But, there exists a theoretical result for response functions in the small noise limit [1], where it has been shown that the CDF of the optimal input distribution, $p_C(X)$, converges to the response function. To check that estimates of $p_C(X)$ from SEMIL satisfies this results, we chose a Hill response function, which is similar used in the original proof [1]. The mean response $\langle g \rangle$ of this function rises from 0 to 1 with increasing input, *X*.

$$\langle g \rangle(X) = \frac{X^n}{X^n + \text{IC}_{50}^n} \tag{12}$$

where $n = 2$ and $\text{IC}_{50} = 50$. We selected a mean-dependent noise function from [1],

$$\sigma^2(\langle g \rangle; \alpha) = \alpha\big(\langle g \rangle + \langle g \rangle^{2-1/n}(1 - \langle g \rangle)^{2+1/n}\big) \tag{13}$$

where the magnitude of the noise for the response function, Eq. (12), scales with α. The conditional output distribution for an input *X* is the normal distribution,

$$p(g|X;\alpha) = \frac{1}{\sqrt{2\pi\sigma^2}} e^{-\frac{(g-\langle g \rangle)^2}{2\sigma^2}} \quad , \tag{14}$$

with $\langle g \rangle$ from Eq. (12) and σ^2 from Eq. (13). While keeping the mean response function, Eq. (13), the same we used $\alpha \in \{1, 0.1, 0.01\}$ to generate output with the same mean but decreasing levels of noise. We selected 20 input concentrations

X = {0.0625, 0.125, 0.25, 0.5, 1, 2, 4, 8, 16, 32, 64, 128, 256, 512, 1024, 2048, 4096, 8192, 16384, 32768}

at each of which we drew 10000 samples from the conditional output distribution Eq. (14) to obtain a set of discretized distributions for $P(g|X;\alpha)$. Then we used SEMIL to compute the optimal input distribution, $p_C(X)$, for each noise level (or α).

4. **EVALUATION OF SEMIL WITH MINIMAL BRN STOCHASTIC SIMULATIONS**

We further evaluated SEMIL using simulated data for a BRN modeled after the *lac* operon from *E. coli*. Using the Gillespie algorithm, we generated simulated output data as described below. We then used the simulation results to compute the mutual information.

To calculate the correct result, we used simulation results for a dense set of input values to numerically integrate the mutual information as:

$$I(X;g) = \int_X p(X)\left[\sum_j P(g = g_j|X)\log_2 \frac{P(g = g_j|X)}{P(g = g_j)}\right] dX\ . \qquad (15)$$

where $P(g = g_j|X)$ is the probability for a cell to contain g_j protein molecules given an input value of X, and $P(g = g_j) = \int_X p(X)P(g=g_j|X)dX$ is the marginal probability for a cell to contain g_j output reporter protein molecules. To estimate $P(g = g_j|X)$ and $P(g = g_j)$ from the simulation results, we directly used the observed frequencies from the simulated data. Note that the Gillespie simulations give a discrete result for the gene expression output (number of protein molecules) so the integration over g in Eq. (11) is replaced by a summation over g_j in Eq. (15). Also, for numerical integration over the domain of X, we divided the domain of $\log_{10}X$ into 400 equal intervals or 401 values of X for which we obtained estimates of the output distribution from independent Gillespie simulations. The correct mutual information landscape computed using numerical integration of Eq. (15) is shown in Supplementary Figure 3.

To estimate the mutual information using SEMIL, we took sparse subsets of the simulated data at 5, 10, and 20 discrete input values and used the corresponding simulated output data with the SEMIL algorithm. Results comparing the SEMIL estimates and the correct result are shown in Fig. 3 in the main text.

The model BRN used for Gillespie simulations is based on a reaction network model of the *lac* operon [2], where an input concentration controls the output response of a protein by interacting with a repressor-operator complex. The input to this BRN is the concentration of isopropyl β-D-1-thiogalactopyranoside (IPTG). The stochastic simulations were done using the Gillespie algorithm. Tables 1-4 list the components of the BRN, the reactions, propensity functions and rate constants.

The domain of the input, X = IPTG concentration, was from 10^{-1} μmolL^{-1} to 10^{4} μmolL^{-1}. We divided the interval $\log_{10}X$ into 400 equal intervals, resulting in 401 input values. We simulated the BRN (Tables 1-4) for these 401 concentrations of IPTG to get the output data used for evaluation. Each simulation was run for 2600 minutes and the first 100 minutes were neglected to remove any effect of the initial condition. The output reporter protein level in the BRN was sampled after every 0.01 minutes. Examples of discrete distributions of the output reporter protein are shown in Supplementary Figure 2.

For the mutual information landscapes with SEMIL we used the following sets of input values, which are roughly equally spaced in the domain of the input $\log_{10} X$.

1. Five input values (Fig. 3(a, d) in the main text): X = {1, 5.6, 31.6, 177.8, 1000} μmolL^{-1}.

2. Ten input values (Fig. 3(b, e) in the main text): X = {0.7, 1.58, 3.55, 7.9, 17.8, 39.8, 89.1, 199.5, 446.7, 1000} μmolL^{-1}.

3. Twenty input values (Fig. 3(c, f) in the main text): X = {0.4, 0.6, 1, 1.58, 2.51, 3.98, 6.5, 10, 15.85, 25.1, 39.8, 63.1, 100, 158.85, 244.06, 398.1, 668.34, 1000, 1631.2, 2585.2} μmolL^{-1}.

Two examples of the set of biased mutual information as a function of N_T /N are shown in Supplementary Figure 4. We have shown the mutual information for larger values of N_T /N in the figure only to demonstrate the effect of the second-order term. But in our work, we calculated the I_{bias} for up to $N_T /N = 20$. For the BRNs with deactivated *lacY* the linear approximation of Eq. (14) up to $N_T /N = 20$ was applicable. But for BRNs with activated *lacY* we could use only up to $N_T /N = 10$ for the linear fit. With the experimentally measured BRN output data used for this work, there were typically more than 5,000 single cell observations for each input value. Consequently, the mutual information estimates obtained directly from the full data ($N_T /N = 1$) were close to the extrapolated, unbiased estimate (within 0.01 bits near the optimal input distribution and typically within 0.1 bits near the boundaries of the design space).

5. MUTUAL INFORMATION LANDSCAPES FOR EXPERIMENTAL BRNS AND OTHER EXPERIMENTAL DETAILS.

Mutual information landscapes of replicate measurements

The mutual information landscapes for the replicate measurements are in Supplementary Figures 5-10. The black or white dots show the coordinates of the optimal input distribution, (E_C,σ_C), and the same colored contours around these points bound the mutual information values that are within 0.05 bits from the maximum mutual information.

Mutual information landscapes using smaller sets of input values

We took one of the replicates for each of the six experimentally-studied BRNs and studied the impact of a smaller set of output data on the mutual information landscape from SEMIL. Supplementary Figures 11-16 show the landscapes by systematically using the output data for a subset of inputs values. The first panel of each of the figures shows the mutual information landscape obtained using one-fourth (5) of the input values, the second panel uses half (9) of the input values, and the third panel uses the total set (18) of input values. The landscapes obtained using the half and the full set of input values are indistinguishable for each of the BRNs. The difference in the mutual information landscapes from 5 and 9 input values is only prominent for BRNs that have a relatively higher maximum mutual information (Supplementary Figure 12-13).

Maximum mutual information from SEMIL and Blahut-Arimoto algorithm

The most common method to determine the maximum mutual information (I_{max}), or the channel capacity, is the Blahut-Arimoto algorithm[7]. We compare the maximum mutual information obtained using Blahut-Arimoto algorithm and SEMIL in Supplementary Figure 17. SEMIL evaluates mutual information constrained to a chosen design space of input probability distributions, whereas Blahut-Arimoto performs an unconstrained search. Hence the maximum mutual information from SEMIL is consistently lower than the estimate from Blahut-Arimoto algorithm. However, the optimal input distribution from Blahut-Arimoto is spiky and discontinuous and difficult to interpret as a biologically plausible input distribution [3], SEMIL

circumvents this problem of interpretation by using a well-defined design space of continuous input distributions.

Details of *E. coli* culture conditions

Details of the growth conditions used for the *E. coli* cultures are provided in Supplementary Table 6 and 7, in the format recommended as a minimum information standard for bacterial cell growth [8].

Plasmid sequence

The plasmid map used for engineered BRNs is shown in Supplementary Figure 18. The complete plasmid sequence with annotations is given below:

CCAATTATTGAAGGCCTCCCTAACGGGGGGCCTTTTTTTGTTTCTGGTCTCCCGCTTAACGATC
GTTGGCTGTGTTGACAATTAATCATCGGCTCGTATAATGTGTGGAATTGTGAGCGCTCACAATT
AGCTGTCACCGGATGTGCTTTCCGGTCTGATGAGTCCGTGAGGACGAAACAGCCTCTACAAATA
ATTTTGTTTAATACTAGAGAAAGAGGGGAAATACTAGATGGTGAGCAAGGGCGAGGAGCTGTTC
ACCGGGGTGGTGCCCATCCTGGTCGAGCTGGACGGCGACGTAAACGGCCACAAGTTCAGCGTGT
CCGGCGAGGGCGAGGGCGATGCCACCTACGGCAAGCTGACCCTGAAGTTCATCTGCACCACAGG
CAAGCTGCCCGTGCCCTGGCCCACCCTCGTGACCACCTTCGGCTACGGCCTGCAATGCTTCGCC
CGCTACCCCGACCACATGAAGCTGCACGACTTCTTCAAGTCCGCCATGCCCGAAGGCTACGTCC
AGGAGCGCACCATCTTCTTCAAGGACGACGGCAACTACAAGACCCGCGCCGAGGTGAAGTTCGA
GGGCGACACCCTGGTGAACCGCATCGAGCTGAAGGGCATCGACTTCAAGGAGGACGGCAACATC
CTGGGGCACAAGCTGGAGTACAACTACAACAGCCACAACGTCTATATCATGGCCGACAAGCAGA
AGAACGGCATCAAGGTGAACTTCAAGATCCGCCACAACATCGAGGACGGCAGCGTGCAGCTCGC
CGACCACTACCAGCAGAACACCCCAATCGGCGACGGCCCCGTGCTGCTGCCCGACAACCACTAC
CTTAGCTACCAGTCCGCCCTGAGCAAAGACCCCAACGAGAAGCGCGATCACATGGTCCTGCTGG
AGTTCGTGACCGCCGCCGGGATCACTCTCGGCATGGACGAGCTGTACAAGTAATAATAACCGGG
CAGGCCATGTCTGCCCGTATTTCGCGTAAGGAAATCCATTATGTACTATTTAAAAAACACAAAC
TTTTGGATGTTCGGTTTATTCTTTTTCTTTTACTTTTTTATCATGGGAGCCTACTTCCCGTTTT
TCCCGATTTGGCTACAT**GACATCAAC**CATATCAGCAAAAGTGATACGGGTATTATTTTTGCCGC
TATTTCTCTGTTCTCGCTATTATTCCAACCGCTGTTTGGTCTGCTTTCTGACAAACTCGGGCTG
CGCAAATACCTGCTGTGGATTATTACCGGCATGTTAGTGATGTTTGCGCCGTTCTTTATTTTTA
TCTTCGGGCCACTGTTACAATACAACATTTTAGTAGGATCGATTGTTGGTGGTATTTATCTAGG
CTTTTGTTTTAACGCCGGTGCGCCAGCAGTAGAGGCATTTATTGAGAAAGTCAGCCGTCGCAGT
AATTTCGAATTTGGTCGCGCGCGGATGTTTGGCTGTGTTGGCTGGGCGCTGTGTGCCTCGATTG
TCGGCATCATGTTCACCATCAATAATCAGTTTGTTTTCTGGCTGGGCTCTGGCTGTGCACTCAT
CCTCGCCGTTTTACTCTTTTTCGCCAAAACGGATGCGCCCTCTTCTGCCACGGTTGCCAATGCG
GTAGGTGCCAACCATTCGGCATTTAGCCTTAAGCTGGCACTGGAACTGTTCAGACAGCCAAAAC
TGTGGTTTTTGTCACTGTATGTTATTGGCGTTTCCTGCACCTACGATGTTTTTGACCAACAGTT
TGCTAATTTCTTTACTTCGTTCTTTGCTACCGGTGAACAGGGTACGCGGGTATTTGGCTACGTA
ACGACAATGGGCGAATTACTTAACGCCTCGATTATGTTCTTTGCGCCACTGATCATTAATCGCA
TCGGTGGGAAAAACGCCCTGCTGCTGGCTGGCACTATTATGTCTGTACGTATTATTGGCTCATC
GTTCGCCACCTCAGCGCTGGAAGTGGTTATTCTGAAAACGCTGCATATGTTTGAAGTACCGTTC

CTGCTGGTGGGCTGCTTTAAATATATTACCAGCCAGTTTGAAGTGCGTTTTTCAGCGACGATTT
ATCTGGTCTGTTTCTGCTTCTTTAAGCAACTGGCGATGATTTTTATGTCTGTACTGGCGGGCAA
TATGTATGAAAGCATCGGTTTCCAGGGCGCTTATCTGGTGCTGGGTCTGGTGGCGCTGGGCTTC
ACCTTAATTTCCGTGTTCACGCTTAGCGGCCCCGGCCCGCTTTCCCTGCTGCGTCGTCAGGTGA
ATGAAGTCGCTTAACTCGGTACCAAATTCCAGAAAAGAGGCCTCCCGAAAGGGGGGCCTTTTTT
CGTTTTGGTCCAATGGCGGCGCGCCATCGAATGGCGCAAAACCTTTCGCGGTATGGCATGATAG
CGCCC**GGAAGAGAGTCAATTCAGGGTGGTGAAT**ATGAAACCAGTAACGTTATACGATGTCGCAG
AGTATGCCGGTGTCTCTTATCAGACCGTTTCCCGCGTGGTGAACCAGGCCAGCCACGTTTCTGC
GAAAACGCGGGAAAAAGTGGAAGCGGCGATGGCGGAGCTGAATTACATTCCCAACCGCGTGGCA
CAACAACTGGCGGGCAAACAGTCGTTGCTGATTGGCGTTGCCACCTCCAGTCTGGCCCTGCACG
CGCCGTCGCAAATTGTCGCGGCGATTAAATCTCGCGCCGATCAACTGGGTGCCAGCGTGGTGGT
GTCGATGGTAGAACGAAGCGGCGTCGAAGCCTGTAAAGCGGCGGTGCACAATCTTCTCGCGCAA
CGCGTCAGTGGGCTGATCATTAACTATCCGCTGGATGACCAGGATGCCATTGCTGTGGAAGCTG
CCTGCACTAATGTTCCGGCGTTATTTCTTGATGTCTCTGACCAGACACCCATCAACAGTATTAT
TTTCTCCCATGAGGACGGTACGCGACTGGGCGTGGAGCATCTGGTCGCATTGGGTCACCAGCAA
ATCGCGCTGTTAGCGGGCCCATTAAGTTCTGTCTCGGCGCGTCTGCGTCTGGCTGGCTGGCATA
AATATCTCACTCGCAATCAAATTCAGCCGATAGCGGAACGGGAAGGCGACTGGAGTGCCATGTC
CGGTTTTCAACAAACCATGCAAATGCTGAATGAGGGCATCGTTCCCACTGCGATGCTGGTTGCC
AACGATCAGATGGCGCTGGGCGCAATGCGCGCCATTACCGAGTCCGGGCTGCGCGTTGGTGCGG
ATATCTCGGTAGTGGGATACGACGATACCGAAGATAGCTCATGTTATATCCCGCCGTTAACCAC
CATCAAACAGGATTTTCGCCTGCTGGGGCAAACCAGCGTGGACCGCTTGCTGCAACTCTCTCAG
GGCCAGGCGGTGAAGGGCAATCAGCTGTTGCCAGTCTCACTGGTGAAAAGAAAAACCACCCTGG
CGCCCAATACGCAAACCGCCTCTCCCCGCGCGTTGGCCGATTCATTAATGCAGCTGGCACGACA
GGTTTCCCGACTGGAAAGCGGGCAGTGATAATCCAGGAGGAAAAAAATGTCCAGATTAGATAAA
AGTAAAGTGATTAACAGCGCATTAGAGCTGCTTAATGAGGTCGGAATCGAAGGTTTAACAACCC
GTAAACTCGCCCAGAAGCTAGGTGTAGAGCAGCCTACATTGTATTGGCATGTAAAAAATAAGCG
GGCTTTGCTCGACGCCTTAGCCATTGAGATGTTAGATAGGCACCATACTCACTTTTGCCCTTTA
GAAGGGGAAAGCTGGCAAGATTTTTTACGTAATAACGCTAAAAGTTTTAGATGTGCTTTACTAA
GTCATCGCGATGGAGCAAAAGTACATTTAGGTACACGGCCTACAGAAAAACAGTATGAAACTCT
CGAAAATCAATTAGCCTTTTTATGCCAACAAGGTTTTTCACTAGAGAATGCATTATATGCACTC
AGCGCTGTGGGGCATTTTACTTTAGGTTGCGTATTGGAAGATCAAGAGCATCAAGTCGCTAAAG
AAGAAAGGGAAACACCTACTACTGATAGTATGCCGCCATTATTACGACAAGCTATCGAATTATT
TGATCACCAAGGTGCAGAGCCAGCCTTCTTATTCGGCCTTGAATTGATCATATGCGGATTAGAA
AAACAACTTAAATGTGAAAGTGGGTCCTAATAATTGGTAACGAATCAGACAATTGACGGCTCGA
GGGAGTAGCATAGGGTTTGCAGAATCCCTGCTTCGTCCATTTGACAGGCACATTATGCATCGAT
GATAAGCTGTCAAACATGAGCAGATCCTCTACGCCGGACGCATCGTGGCCGGCATCACCGGCGC
CACAGGTGCGGTTGCTGGCGCCTATATCGCCGACATCACCGATGGGGAAGATCGGGCTCGCCAC
TTCGGGCTCATGAGCAAATATTTTATCTGAGGTGCTTCCTCGCTCACTGACTCGCTGCACGAGG
CAGACCTCAGCGCTAGCGGAGTGTATACTGGCTTACTATGTTGGCACTGATGAGGGTGTCAGTG
AAGTGCTTCATGTGGCAGGAGAAAAAAGGCTGCACCGGTGCGTCAGCAGAATATGTGATACAGG
ATATATTCCGCTTCCTCGCTCACTGACTCGCTACGCTCGGTCGTTCGACTGCGGCGAGCGGAAA
TGGCTTACGAACGGGGCGGAGATTTCCTGGAAGATGCCAGGAAGATACTTAACAGGGAAGTGAG
AGGGCCGCGGCAAAGCCGTTTTTCCATAGGCTCCGCCCCCCTGACAAGCATCACGAAATCTGAC
GCTCAAATCAGTGGTGGCGAAACCCGACAGGACTATAAAGATACCAGGCGTTTCCCCTGGCGGC
TCCCTCGTGCGCTCTCCTGTTCCTGCCTTTCGGTTTACCGGTGTCATTCCGCTGTTATGGCCGC
GTTTGTCTCATTCCACGCCTGACACTCAGTTCCGGGTAGGCAGTTCGCTCCAAGCTGGACTGTA
TGCACGAACCCCCCGTTCAGTCCGACCGCTGCGCCTTATCCGGTAACTATCGTCTTGAGTCCAA

```
CCCGGAAAGACATGCAAAAGCACCACTGGCAGCAGCCACTGGTAATTGATTTAGAGGAGTTAGT
CTTGAAGTCATGCGCCGGTTAAGGCTAAACTGAAAGGACAAGTTTTGGTGACTGCGCTCCTCCA
AGCCAGTTACCTCGGTTCAAAGAGTTGGTAGCTCAGAGAACCTTCGAAAAACCGCCCTGCAAGG
CGGTTTTTTCGTTTTCAGAGCAAGAGATTACGCGCAGACCAAAACGATCTCAAGAAGATCATCT
TATTAAGGGGTCTGACGCTCAGTGGAACGAAAAATCAATCTAAAGTATATATGAGTAAACTTGG
TCTGACAGTTACCTTAGAAAAACTCATCGAGCATCAAATGAAACTGCAATTTATTCATATCAGG
ATTATCAATACCATATTTTTGAAAAAGCCGTTTCTGTAATGAAGGAGAAAACTCACCGAGGCAG
TTCCATAGGATGGCAAGATCCTGGTATCGGTCTGCGATTCCGACTCGTCCAACATCAATACAAC
CTATTAATTTCCCCTCGTCAAAAATAAGGTTATCAAGTGAGAAATCACCATGAGTGACGACTGA
ATCCGGTGAGAATGGCAAAAGCTTATGCATTTCTTTCCAGACTTGTTCAACAGGCCAGCCATTA
CGCTCGTCATCAAAATCACTCGCATCAACCAAACCGTTATTCATTCGTGATTGCGCCTGAGCGA
GACGAAATACGCGATCGCTGTTAAAAGGACAATTACAAACAGGAATCGAATGCAACCGGCGCAG
GAACACTGCCAGCGCATCAACAATATTTTCACCTGAATCAGGATATTCTTCTAATACCTGGAAT
GCTGTTTTCCCGGGGATCGCAGTGGTGAGTAACCATGCATCATCAGGAGTACGGATAAAATGCT
TGATGGTCGGAAGAGGCATAAATTCCGTCAGCCAGTTTAGTCTGACCATCTCATCTGTAACATC
ATTGGCAACGCTACCTTTGCCATGTTTCAGAAACAACTCTGGCGCATCGGGCTTCCCATACAAT
CGATAGATTGTCGCACCTGATTGCCCGACATTATCGCGAGCCCATTTATACCCATATAAATCAG
CATCCATGTTGGAATTTAATCGCGGCCTCGAGCAAGACGTTTCCCGTTGAATATGGCTCATAAC
ACCCCTTGTATTACTGTTTATGTAAGCAGACAGTTTTATTGTTCATGATGATATATTTTTATCT
TGTGCAATGTACATCAGAGATTTTGAGACACAA
```

In the plasmid sequence given above, the YFP coding DNA sequence (CDS) is highlighted in yellow. The *lacY* CDS is highlighted in cyan. The *lacI* CDS is highlighted in orange. The p15A origin of replication is highlighted in gray. The kanamycin resistance CDS is underlined.

For data shown in the main text Fig. 4, data without feedback (panels b, c, d) was collected by truncating the *lacI* protein at positions D37, I38, and N39 (highlighted cyan, red text) by substituting three stop codons: **TAATAATAG**.

For main text Fig. 4, RBS of *lacI* was changed to modify translation rate of *lacI*. RBS sequence in magenta text above and changed to sequences in Supplementary Table 5.

Flow Cytometry Gating

After growth, 20 µL from each *E. coli* sample was diluted into 180 µL of phosphate buffered saline supplemented with 170 µg/mL chloramphenicol to halt protein translation. The resulting diluted samples were measured on an Attune NxT flow cytometer with 488 nm excitation laser and a 530 19 nm ± 15 nm bandpass emission filter. Blank samples were measured with each set of *E. coli* samples, and the results of the blank measurements were used with an automated gating algorithm to discriminate cell events from non-cell events (Supplementary Figure 19(a,b)). A second automated gating algorithm was used to select singlet cell events and exclude doublet, triplet, and higher-order multiplet cell events. All subsequent analysis was performed using the singlet cell event data (Supplementary Figure 19(a,b)). Between 1,000 and 50,000 singlet cell events were detected and analyzed for each *E. coli* sample.

6. **CORRECTING FOR FINITE-SAMPLING BIAS IN MUTUAL INFORMATION ESTIMATES**

Mutual information estimates obtained directly from data are typically biased. There are two sources of related, but competing bias: First, finite sampling of the data used to estimate probabilities results in a bias of the mutual information estimate toward values that are higher than the true value [3–5]. Second, binning of the data can alleviate some of the finite-sampling bias, but if the number of bins used is too small, the estimated mutual information will be less than the true value [3, 4].

We can use existing methods[3–5] to correct for the finite sampling bias by extrapolating to an infinite amount of data as described below. However, the bias from binning of the data represents an actual loss of information that cannot be recovered. So, the general approach is to choose a number of bins that is sufficiently large to avoid systematic underestimation of the mutual information, but that is also sufficiently small to avoid severe undersampling[6].

We chose the number of bins for the experimentally studied BRNs by estimating the maximum mutual information (without correcting for finite sampling bias) in the design space using different numbers of bins, $nb \in \{100, 250, 500, 1000\}$. We found that the maximum mutual information was independent of the number of bins (constant to within 0.05 bits) for $nb \geq 250$. We therefore selected $nb = 500$ to determine the discretized output distributions $P(g = g_j \mid X = x_i)$ for the BRNs in Supplementary Figure 4.

For the model BRN in Supplementary Figure 1b-1d, we used 100 bins for the output distributions. We repeated the SEMIL calculations with 200 bins and did not observe a significant difference in the mutual information values.

For each mutual information estimate, we corrected for finite sampling bias by extrapolating to an infinite amount of data using previously described methods. The biased mutual information, I_{bias}, is related to the correct mutual information I_∞ by the asymptotic relationship [3, 4]:

$$I_{bias} = I_\infty + \frac{a_1}{N} + \frac{a_2}{N^2} + \ldots \tag{16}$$

where N is the number of samples used to estimate the mutual information. For sufficiently large sample size, N, the second order term in Eq. (16) is negligible and I_{bias} is close a to linear function of $1/N$. Let the total number of available samples be N_T and rewrite Eq. (16) as

$$I_{bias} = I_\infty + \frac{a_1}{N_T}\frac{N_T}{N}, \tag{17}$$

where N_T is the total number of samples available and $N_T/N = 0$ corresponds to infinite samples. We chose subsample sizes, $N_T/N = \{1, 2, 5, 10, 20\}$ and for each value of N_T/N we drew 5 replicate sub-samplings (with replacement) of the output data and computed the corresponding biased estimates of mutual information. We then fit the (I_{bias} , N_T/N) dataset with Eq. (17) using linear

least squares to obtain the unbiased mutual information, I_∞ . This procedure was followed for every point on the design space.

Supplementary References

[1] Warner, J. E., Grigoriu, M. & Aquino, W. Probabilistic Engineering Mechanics 31, 1-11 (2013).
[2] Stamatakis, M. & Mantzaris, N. V. Biophysical journal 96, 887-906 (2009).
[3] Cheong, R., Rhee, A., Wang, C. J., Nemenman, I. & Levchenko, A. Science 334, 354-358 (2011).
[4] Rhee, A., Cheong, R. & Levchenko, A. Physical biology 9, 045011 (2012).
[5] Bialek, W. Biophysics: searching for principles (Princeton University Press, 2012).
[6] Nemenman, I., Lewen, G. D., Bialek, W. & Van Steveninck, R. R. D. R. PLoS computational biology 4, e1000025 (2008).
[7] Blahut, R. IEEE transactions on Information Theory 18.4 (1972).
[8] Hecht, A., Filliben, J., Munro, S. A. & Salit, M. Communications biology 1, 219 (2018).
