# Supplementary material for: Sparse estimation of mutual information landscapes quantifies information transmission through cellular biochemical reaction networks
Source: Commun Biol. 2020 Apr 30;3:203. doi: 10.1038/s42003-020-0901-9 (PMC7192899; doi:10.1038/s42003-020-0901-9)
Supplement: Supplementary file 2 — Description of Additional Supplementary Files [file 42003_2020_901_MOESM2_ESM.pdf]

Description of additional supplementary items

Supplementary Data 1: Data for manuscript Figures 2a-2f.

Supplementary Data 2: Data for manuscript Figures 3a-3f.

Supplementary Data 3: Data for manuscript Figures 4b-4g.
